# Testing the Additivity Assumption: Chemical Mixtures and Thyroid Function

**Published:** 2005-11

**Authors:** Valerie J. Brown

It is well established that many environmental contaminants can disrupt thyroid hormone (TH) homeostasis, which is vital during fetal development and for a variety of physiological processes in adults. Among known TH disruptors are polychlorinated biphenyls (PCBs), dioxins, and dibenzofurans, all members of the polyhalogenated aromatic hydrocarbon (PHAH) chemical family. Little is known, however, about how mixtures of such chemicals at typical environmental exposure levels may disrupt TH functions. Nor is it clear whether effects are additive, synergistic, or antagonistic—that is, whether there is interaction between constituent chemicals, whether their cumulative influence is more than the sum of its parts, or whether they cancel each other out. With respect to risk assessment, the U.S. Environmental Protection Agency’s default assumption is that the effects of chemicals in mixtures are additive. Now a team of researchers has tested the additivity assumption and found that it is relatively robust at exposure levels typical for humans **[*EHP* 113:1549–1554]**.

Over a four-day period the team exposed young female rats to six different doses of a combination of 18 PHAHs comprising 2 dioxins, 4 dibenzofurans, and 12 PCBs. The team determined dose–response information for each constituent chemical before the mixture was tested. The concentration of each chemical in the mixture reflected typical concentrations measured in breast milk and in fish and other foods. The mixture was also formulated so that even at the highest mixture doses, the rats’ exposure to each constituent chemical was at or below the known no-observed-effect level for that chemical.

The mixture reduced the rats’ serum thyroxine (T_4_; the most common form of circulating TH) in a dose-dependent manner. At lower doses the effects were additive. At higher doses T_4_ declined by as much as 50%, and the effects were mildly synergistic—about twice what was predicted by additivity—so that even in the upper range the effects as predicted by the additivity hypothesis came close to actual results.

Significantly, the study also showed that the mixture exerted an effect on T_4_ even though concentrations of its constituent chemicals were at least an order of magnitude below their known effective doses. This indicates that considering individual chemicals in isolation may not predict their effects in mixtures because, even though chemicals may not be potent enough by themselves to cause effects, the cumulative effects of low doses of many chemicals may be enough to do so.

The multiple functions of TH, such as its role in fetal development and its regulation of metabolism and heart rate, make it vulnerable at many points. The team estimates that there could be as many as five distinct mechanisms by which chemicals exert antithyroid effects for which a reduction in circulating T_4_ is the common end point.

Several factors temper the study results. One is that this study was a series of short-term exposures that did not encompass all the chemicals’ varied half-lives. The results therefore cannot be directly extrapolated to the effects of chronic exposures and may be subject to confounding by pharmacokinetic differences. Another is that thyroid disruption mechanisms in rats may not be identical to those in humans. The team is now working on testing how a more complex chemical mixture may interact with dietary iodine insufficiency to produce thyrotoxic effects.

